# Infant Perioperative Risk Factors and Adverse Brain Findings Following Long-Gap Esophageal Atresia Repair

**DOI:** 10.3390/jcm12051807

**Published:** 2023-02-23

**Authors:** Mackenzie Shea Kagan, Jue Teresa Wang, Danielle Bennett Pier, David Zurakowski, Russell William Jennings, Dusica Bajic

**Affiliations:** 1 Department of Anesthesiology, Critical Care and Pain Medicine, Boston Children’s Hospital, 300 Longwood Avenue, Bader 3, Boston, MA 02115, USA; 2 Department of Anaesthesia, Harvard Medical School, 25 Shattuck Street, Boston, MA 02115, USA; 3 Department of Neurology, Division of Pediatric Neurology, Massachusetts General Hospital, 55 Fruit Street, Wang 708, Boston, MA 021114, USA; 4 Department of Surgery, Esophageal and Airway Treatment Center, Boston Children’s Hospital, 300 Longwood Avenue, Boston, MA 02115, USA

**Keywords:** association analysis, critical care, correlations, LGEA, MRI, neurology, pediatrics

## Abstract

Recent findings implicate brain vulnerability following long-gap esophageal atresia (LGEA) repair. We explored the relationship between easily quantifiable clinical measures and previously reported brain findings in a pilot cohort of infants following LGEA repair. MRI measures (number of qualitative brain findings; normalized brain and corpus callosum volumes) were previously reported in term-born and early-to-late premature infants (n = 13/group) <1 year following LGEA repair with the Foker process. The severity of underlying disease was classified by an (1) American Society of Anesthesiologist (ASA) physical status and (2) Pediatric Risk Assessment (PRAm) scores. Additional clinical end-point measures included: anesthesia exposure (number of events; cumulative **m**inimal **a**lveolar **c**oncentration (MAC) exposure in hours), length (in days) of postoperative intubated sedation, paralysis, antibiotic, steroid, and total parenteral nutrition (TPN) treatment. Associations between clinical end-point measures and brain MRI data were tested using Spearman rho and multivariable linear regression. Premature infants were more critically ill per ASA scores, which showed a positive association with the number of cranial MRI findings. Clinical end-point measures *together* significantly predicted the number of cranial MRI findings for both term-born and premature infant groups, but none of the individual clinical measures did on their own. Listed easily quantifiable clinical end-point measures could be used *together* as indirect markers in assessing the risk of brain abnormalities following LGEA repair.

## 1. Introduction

Esophageal atresia, although a rare congenital anomaly with a stable prevalence around the world [1], is one of the most common gastrointestinal birth defects, with a reported incidence of 1 in 2500 to 1 in 4500 live births [2,3]. Compared to short-gap esophageal atresia, long-gap esophageal atresia (LGEA) is more likely to be an isolated defect and associated with Trisomy 21 [4], but is less commonly associated with other anomalies (viz. VACTERL or CHARGE syndromes) [4,5]. Unlike short-gap defects that can be repaired by direct anastomosis (requiring one major surgery and postoperative pain treatment within 5 days) [6], long disconnects (>3 cm) found in LGEA [4] require more complex treatment. At our institution, infants born with LGEA undergo tension-induced esophageal growth as part of the Foker process repair [7,8,9]. The Foker process allows for growth and lengthening of infant’s existing esophageal pouches, spanning a period of weeks [10,11], but requires at least two separate thoracotomies/thoracoscopies [12] before direct anastomosis is achieved. The unique aspect of such complex repair involves not only repeated anesthesia exposure, but prolonged sedation of ≥5 days that is known to be associated with the development of tolerance and physical dependence to the drugs of sedation [13,14,15].

The impact of complex perioperative care with the Foker process on neurobehavioral outcomes in infants born with LGEA represents a major gap in our knowledge. Recent reports indicate that infants undergoing neonatal surgery for noncardiac congenital anomalies, including those with esophageal atresia, are at risk of brain injury [10,16], potentially accounting for the neurodevelopmental delay observed in populations of infants with gastrointestinal anomalies [17]. Our recent pilot studies using brain magnetic resonance imaging (MRI) [10,11,18,19,20] have provoked concerns over the impact of complex perioperative critical care with the Foker process on brain findings and brain growth in infants born with LGEA [10,18,20,21].

Therefore, the main objective in this novel report was to analyze associations between easily quantifiable clinical measures and previously reported brain findings in a pilot cohort of infants that underwent research brain MRI following Foker process repair for LGEA [10,19,20]. In this study, we hypothesized that either higher clinical severity scores (viz. American Society of Anesthesiologist (ASA) [22] and recently introduced Pediatric Risk Assessment measure (PRAm) [23]), or longer exposure to anesthesia and drug treatment (viz. length of postoperative intubated sedation, muscle relaxants, antibiotics, steroids), and/or TPN administration, would be (1) positively associated with the number of incidental brain MRI findings (novel data) and (2) negatively associated with previously published normative total brain [10,20] and corpus callosum volumes [19]. Since our pilot cohort included infants that underwent research brain MRI in the first year of life following repair of LGEA, our secondary objective was to demonstrate association trends with age.

## 2. Methods

### 2.1. Study Design and Participants

This work builds on our previous work (2015–2018) of brain measure quantification using structural magnetic resonance imaging (MRI) [10,19,20] and was approved by the Institutional Review Board as a ‘no more than minimal risk’ study (IRB-P000007855). Informed written parental consent was obtained prior to subject participation, in accordance with the Declaration of Helsinki and Good Clinical Practice guidelines. We previously described a detailed methodological approach for (1) recruitment criteria and (2) scanning process for research brain MRI [10].

In this cross-sectional pilot study, infants’ eligibility criteria included term-born (37 to 42 weeks gestational age at birth) and early-to-late premature infants <1 year gestation-corrected age (n = 13/group) following LGEA repair with Foker process [7,8,9], who developed dependency to drugs of sedation (e.g., opioids and benzodiazepines) [15]. The preterm group included only very preterm (28 to <32 weeks GA) and moderate-to-late preterm infants (32 to <37 weeks GA), as defined by *The World Health Organization* [24]. All infants underwent *external* traction as part of the Foker process that requires infants to stay intubated, muscle relaxed, and sedated postoperatively [25,26]. We did not analyze: (i) post-operative drug sedation management, as such treatment is not protocolized at our institution, or (ii) potential symptoms of withdrawal (see [27] for review on weaning). Instead, we confirmed that weaning management to drugs of sedation occurred as per primary team and/or pain service notes. Exclusion criteria were: (1) extreme prematurity (<28 weeks GA); (2) diagnosis of small for gestational age and/or intrauterine growth restriction (SGA/IUGR) [28,29]; (3) history of cardiac arrest and/or major cardiovascular resuscitation; (4) extracorporeal membrane oxygenation exposure; (5) status post tracheostomy; (6) clinically indicated cranial ultrasound findings (e.g., ventricular enlargement with or without intraparenchymal and/or intraventricular hemorrhage); (7) neurological disease as documented in clinical record (e.g., seizures, craniosynostosis requiring surgical repair); (8) chromosomal abnormalities (e.g., Down’s syndrome); (9) prenatal drug exposure to either drugs of abuse or prescription medications; and/or (10) MRI-incompatible implants. Our pilot cohort [30] included infants with isolated LGEA and LGEA with tracheo-esophageal fistula (TEF), while a few patients had other non-cardiac congenital anomaly diagnoses that included LGEA as part of VACTERL association (without a cardiac component). None of the cohort infants had cardiac anomalies requiring surgery, nor exposure to extracorporeal membrane oxygenation, and had no clinical evidence of neurological problems at the time of recruitment, as per detailed chart review and/or cranial ultrasound findings when available (n = 13/group) [30].

### 2.2. Brain MRI Acquisition and Structural Analyses

Structural research brain MRI was obtained under natural sleep according to the ‘feed and wrap’ approach. All infants were scanned either just before hospital discharge following Foker process [7,8,9] (including completion of weaning) or during subsequent admissions for follow-up management in the 1st year of life. Thus, subjects were scanned at different times during gestation-corrected first year of life in relation to the completion of treatment, depending on the time of recruitment. Structural T1- and T2-weighted MRI data were successfully collected for all subjects, allowing for detailed qualitative and quantitative data analysis for term-born and premature groups (n = 13/group). A pediatric neuroradiologist on call reviewed MRI scans for any findings of clinical significance. Additionally, a pediatric neurologist blindly evaluated the same (DBP). Clinically relevant MRI findings included those related to extra-cranial (e.g., abnormal head shape) and intra-cranial findings (e.g., increased extra-axial space, ventriculomegaly, thinning of corpus callosum, subdural hematoma, stroke, etc. [10,18,19]). This qualitative evaluation was summed as the individual total number of cranial MRI findings (novel data). Please refer to our previous reports for detailed description of protocols for (i) preparation and supervision of infants undergoing non-sedated brain MRI [10], (ii) details of structural scanning parameters [10,20], (iii) quantitative T2-weighted brain segmentation [10,20] using Morphologically Adaptive Neonatal Tissue Segmentation (MANTiS) toolbox [31], as well as (iv) quantitative T1-weighted brain and corpus callosum [19,30] volume estimation, since it is beyond the scope of this manuscript. As previously published, we reported normalized volumes of the brain as % of intracranial volume [10,21], and that of corpus callosum as % of total forebrain volume [19].

### 2.3. Underlying Disease Severity Scores and Clinical Parameter Acquisition

In addition to demographic information (see Table 1 in [30]), several clinical end-point measures were obtained from the electronic medical records (PowerChart^®^, Cerner, London, UK) and digital anesthesia records (AIMS Charts, 2019 Citrix Receiver Application, v. 19.3.0.21) for each patient.

#### 2.3.1. Disease Severity Scores

We collected 2 underlying disease severity scores used in clinical practice to assess underlying disease complexity: (1) American Society of Anesthesiologists (ASA) physical status classifications score [22], and (2) Pediatric Risk Assessment (PRAm) score [23]. According to the ASA physical status classification, infants are rated on a scale of I (healthy) to VI (braindead). We collected the highest individual ASA score value, as documented by anesthesia charts at the time of initial esophageal repair surgery (Foker I).

In contrast to ASA, PRAm score was most recently introduced as a novel score to predict perioperative mortality in children undergoing noncardiac surgery [23]. Thus, our research team calculated PRAm scores for infants recruited before 2017. Scoring of PRAm involves the following: urgency of surgical procedure (+1), presence of at least one comorbidity (+2), presence of at least one indication of critical illness (+3), age < 12 months at surgery (+3), and co-existing malignancy (+4) for a range of scores from 0 to 13, with 0 representing the least threat to life and 13 representing the greatest threat to life. Since all infants in this pilot cohort were younger than 12 months, and had no co-existing malignancy, PRAm scores ranged from score 3 to 9.

#### 2.3.2. Clinical Care Measures

We collected 7 easily quantifiable clinical end-point measures from medical records: (1) number of anesthesia events, and (2) cumulative anesthesia exposure as total minimal alveolar concentration (MAC)-equivalent anesthesia hours up to the time of the research brain MRI. All infants received inhalational agents for maintenance of anesthesia during all procedures. None of the infants underwent total intravenous anesthesia (TIVA) for any procedure. Details of anesthesia management (e.g., administration of inhalational agents with/without peripheral nerve block and/or intravenous pain management) are beyond the subject of this manuscript. We also quantified duration of postoperative treatment (in days) of (3) mechanical ventilation and (4) muscle relaxation (as indirect measures of sedation), as well as (5) antibiotics, (6) steroids, and (7) total parenteral nutrition (TPN) with fat emulsion as an indirect measure of complexity of postoperative care (e.g., surgical complications). All listed data metrics were collected from the perioperative period up until the time of research brain MRI scan. Sedation and weaning management were administered as infusions with boluses. It included combination of opioid (e.g., morphine), benzodiazepine (e.g., midazolam), hypnotic (e.g., ketamine), and/or alpha2-agonist (e.g., dexmedetomidine). Neither sedation nor post-operative muscle relaxation was protocolized. For the few infants that underwent minor procedures at other institutions prior to transfer to our institution for esophageal repair, duration of anesthesia events was estimated (i.e., endotracheal intubation = 0.25 h; placement of central access line = 0.5 h; laparoscopic gastrostomy placement = 1 h), likely resulting in the underestimation of total anesthesia exposure. Due to incomplete availability of outside hospital documentation, comprehensive postoperative intubated sedation/muscle relaxation data were compiled for n = 11/13 term-born infants and n = 10/13 premature infants. Additionally, antibiotic, steroid, and TPN exposure data were only available for n = 12/13 infants per either group. We eliminated only one term-born data point at 12 months of age from correlation analysis as it was considered an aberrant point. This subject underwent extraordinarily long TPN and antibiotic administration that artificially diverted the statistics.

### 2.4. Statistical Analysis

As this was a pilot MRI study [10] and no prior information was available regarding brain volumes in the selected cohort of infants with LGEA, a convenience sample size of 13 infants/group was chosen, based on the anticipated number of eligible infants at our institution and an estimated 50% enrollment rate. Statistical analysis was performed using the Statistical Package for the Social Sciences (SPSS, v.23.0; IBM Corporation, Armonk, NY, USA).

#### 2.4.1. Correlation Analysis

The Shapiro–Wilk test was used to assess for normality of data, while associations were determined by nonparametric Spearman Rho (r) test, which is resistant to the effects of outliers [32]. Strength of correlation was described as: weak (r < 0.4), moderate (r ≥ 0.4 to <0.7), and strong (r ≥ 0.7) [33]. We used more stringent Bonferroni criteria for *p* < 0.01 as statistically significant as to protect against false-positive results due to the multitude of testing [34].

#### 2.4.2. Multivariable Linear Regression

A multivariable linear regression model was used to identify independent predictors of adverse neurological data, as quantified by research brain MRI. After testing for multicollinearity using variance inflation factor (VIF) measures, the following 6 variables were included in the final model: (1) group status, (2) cumulative MAC-equivalent hours of anesthesia exposure, and length of (3) postoperative mechanical ventilation, (4) postoperative muscle relaxation, (5) antibiotic, and (6) steroid administration. TPN was excluded from the final model due to its correlation with the other clinical variables. Multivariable linear regression results are presented as B coefficients with 95% confidence intervals and *p* values. A two-tailed a level of <0.05 was used to assess for statistical significance.

## 3. Results

### 3.1. Brain MRI Measures

**Figure 1:**
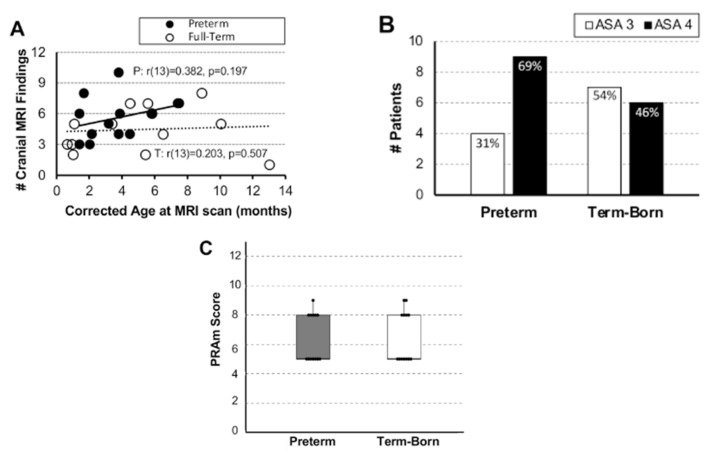
**Incidence of Qualitative Brain Findings per Patient and Underlying Disease Severity Scores.** Panel (**A**) graph shows no significant associations between individual number of cranial MRI findings and age at the time of research brain MRI scan for early-to-late preterm (n = 13; black circles, solid trend line) and term-born infants (n = 13; open circles, dashed trend line) who underwent Foker process [7] for long-gap esophageal atresia (LGEA) repair. Panels (**B**,**C**) show distribution of American Society of Anesthesiologist (ASA) classification and Pediatric Risk Assessment (PRAm) scores, respectively. The percent (%) of infants per gestational age group for ASA score is shown for each bar (Panel (**B**)). In contrast, PRAm scoring is rated on a wider scale of 0–13, representing the least and the greatest threat to life, respectively. Dots in Panel (**C**) represent individual scores, boxes span the interquartile range (IQR) (first and third quartile), and whiskers represent maximum and minimum values. *Abbreviations*: **#**, number; P, premature; T, term-born.

In this report, we summed qualitative cranial and brain findings of clinical significance that included findings of the gray and white brain matter, as well as vascular abnormalities. Irrespective of the gestation age groups, the most frequent qualitative brain findings (Table 1) were: (1) abnormalities of corpus callosum (viz. shape, size, and hypomyelination), (2) enlarged/prominent ventricles, and (3) increased extra-axial fluid. We report no significant association between the individual number of cranial MRI findings and age (Figure 1A).

### 3.2. Underlying Disease Severity: American Society of Anesthesiology (ASA) Classification System and Pediatric Risk Assessment (PRAm) Scoring

#### 3.2.1. Disease Severity Score Distribution

We summarize disease severity scores (ASA physical status classification and PRAm scoring) of the pilot cohort based on the gestation age. As expected, prematurity is associated with higher ASA score of IV (9/13; 69%), while term-born infants have an equal distribution between ASA scores of III and IV (Figure 1B). Despite wider scoring range for PRAm (3–9), cohort patients had a similar distribution across a 5–9 score range, irrespective of the gestational age group (Figure 1C).

#### 3.2.2. Associations between Disease Severity Scores and Brain MRI Data

**Figure 2:**
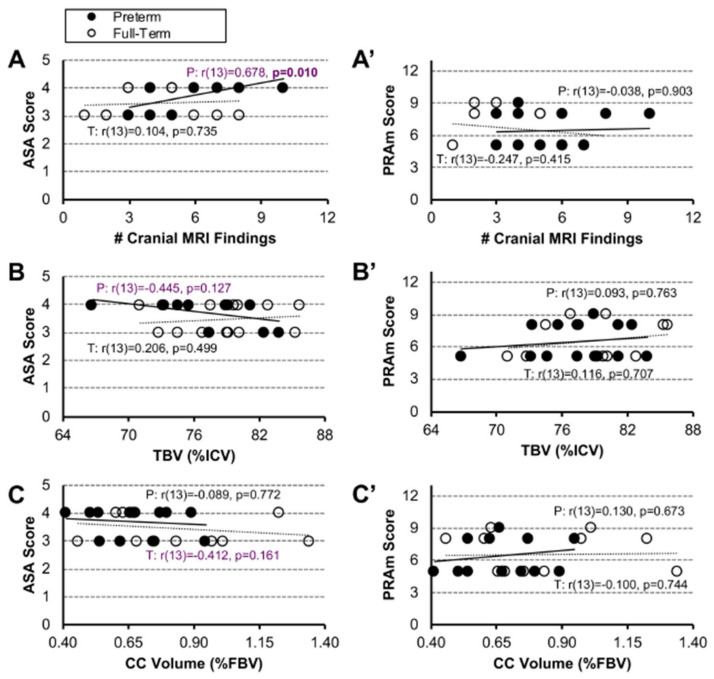
**Association Between Disease Severity Scores and Brain MRI Metrics.** Associations between an American Society of Anesthesiologist (ASA) classification (**A**–**C**) or Pediatric Risk Assessment (PRAm) scores (**A′**–**C′**) with 3 different brain MRI metrics for early-to-late preterm (n = 10–13; black circles, solid trend line) and term-born infants (n = 11–13; open circles, dashed trend line) following long-gap esophageal atresia (LGEA) repair. Neither group showed significant associations. All correlations were assessed by nonparametric Spearman Rho tests, which are resistant to the effects of outliers. Strength of correlation is described as weak (r < 0.4; black), moderate (r ≥ 0.4 to <0.7; purple), or strong, (r ≥ 0.7; red) with statistical significance as *p* < 0.01 (2-tailed). *Abbreviations*: #, number; **%**, percent; CC, corpus callosum; FBV, forebrain volume; ICV, intracranial volume; P, premature; T, term-born; TBV, total brain volume.

Despite moderate associations between ASA scores and brain MRI metrics in early-to-late preterm infants, we did not show any significant relationship in either preterm or term-born infant patients (Figure 2A–C). Similarly, there is no association between PRAm scores and cranial MRI findings, irrespective of the gestational age group (Figure 2A′–C′).

### 3.3. Quantification of Clinical Measures of Care

We quantified seven clinical endpoint measures, which included (I) measures of anesthesia exposure (number of anesthesia events and MAC-equivalent cumulative anesthesia hours), (II) indirect measures of postoperative sedation (viz. length of postoperative mechanical ventilation and muscle relaxation in days), as well as (III) indirect measures of postoperative surgical complications and care (viz. length of antibiotics, steroids, and TPN administration in days) up to the time-point of research brain MRI.

#### 3.3.1. Associations between Clinical Measures and Age

**Figure 3:**
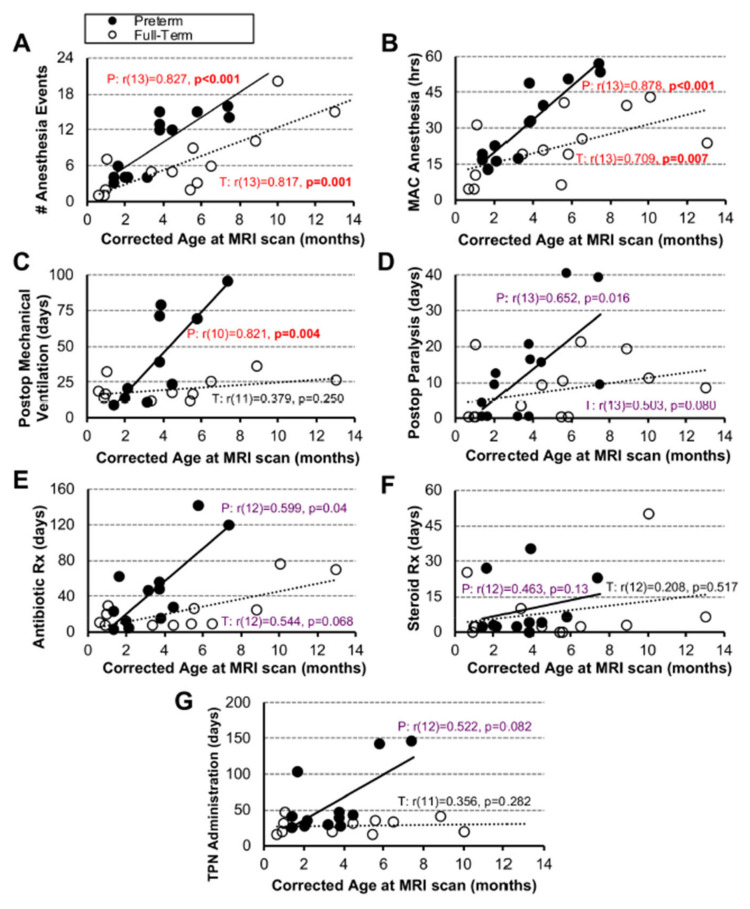
**Association Between Clinical Measures and Age.** Panels (**A**–**G**) show associations between 7 different clinical care measures and age for early-to-late preterm (n = 10–13; black circles, solid trend line) and term-born infants (n = 11–13; open circles, dashed trend line) following long-gap esophageal atresia (LGEA) repair. All correlations were assessed by nonparametric Spearman Rho tests, which are resistant to the effects of outliers. Data were non-normally distributed for clinical measures in Panels (**D**–**G**). Strength of correlation is described as weak (r < 0.4; black), moderate (r ≥ 0.4 to <0.7; purple), or strong (r ≥ 0.7; red) with statistical significance as *p* < 0.01 (2-tailed). *Abbreviations*: **#**, number; MAC, minimal alveolar concentration; P, premature; Postop, post-operative; Rx, treatment; T, term-born; TPN, total parenteral nutrition.

Since our pilot cohort included infants that underwent research brain MRI in the first year of life following repair of LGEA with the Foker process, we investigated the relationship between each clinical parameter with age at the time of brain MRI scan. We report a positive association between all clinical measures with age (Figure 3). Specifically, anesthesia exposure (viz. number of anesthesia events and cumulative MAC hours) shows significant positive associations in both premature (# anesthesia events: r(13) = 0.827, *p* < 0.001; cumulative MAC hours: r(13) = 0.878, *p* < 0.001), and term-born infants (# anesthesia events: r(13) = 0.817, *p* = 0.001; cumulative MAC hours: r(13) = 0.709, *p* = 0.007) with age (Figure 3A,B). This reflects repeated anesthesia exposure in infancy (e.g., revisions or follow-up esophagoduodenoscopies). We also report a significant positive association only in premature infants for length of postoperative mechanical ventilation with age (r(10) = 0.821, *p* = 0.004), while other clinical measures did not show any significant associations with age (Figure 3D–G).

#### 3.3.2. Associations between Clinical Measures and Disease Severity Scores

In the selected cohort of early-to-late premature and term-born infants following LGEA repair with the Foker process (n = 13/group), we did not find any significant associations between any of the clinical end-point measures with either ASA or PRAm scores, irrespective of the gestational age.

#### 3.3.3. Associations between Clinical Measures and Brain MRI Measures

**Figure 4:**
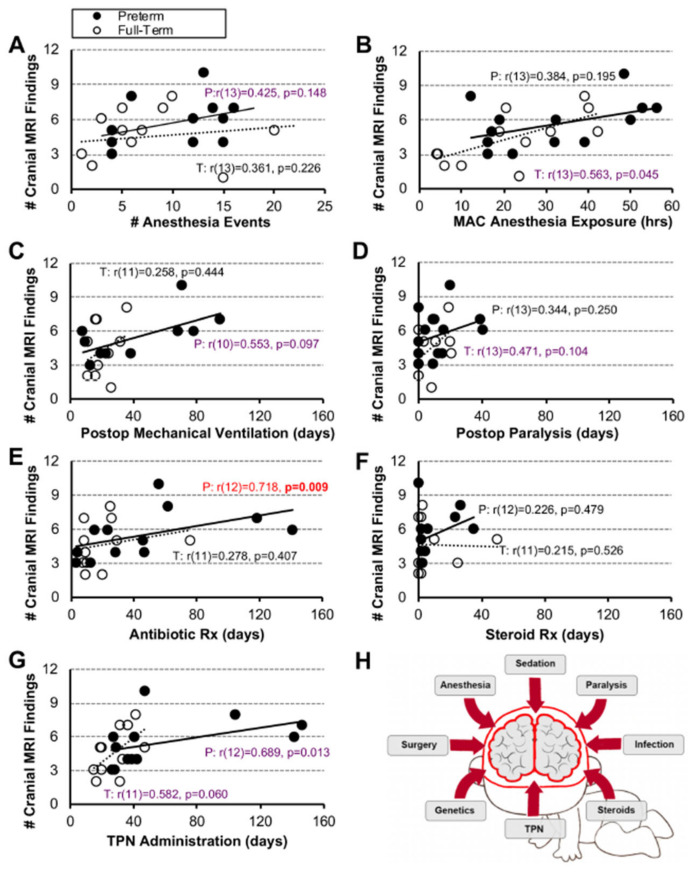
**Association Between Clinical Measures and Number of Cranial MRI Findings**. Associations between 7 clinical care measures and the number (#) of cranial MRI findings (Panels (**A**–**H**) for early-to-late preterm (n = 10–13; black circles, solid trend line) and term-born infants (n = 11–13; open circles, dashed trend line) following long-gap esophageal atresia (LGEA) repair. All correlations were assessed by nonparametric Spearman Rho tests, which are resistant to the effects of outliers. Data were non-normally distributed for clinical measures in Panels (**D**–**G**). Strength of correlation is described as weak (r < 0.4; black), moderate (r ≥ 0.4 to <0.7; purple), or strong, (r ≥ 0.7; red) with statistical significance as *p* < 0.01 (2-tailed). *Abbreviations*: **#**, number; MAC, minimal alveolar concentration; P, premature; Postop, post-operative; Rx, treatment; T, term-born; TPN, total parenteral nutrition.

**Number of Cranial MRI Findings.** As illustrated in Figure 4, we report a significant positive association only between the length of antibiotic treatment and the number of cranial MRI findings in premature infants (r(12) = 0.718, *p* = 0.009; Figure 4E). We did not find any significant positive association between listed measures for the term-born infants.

**Figure 5:**
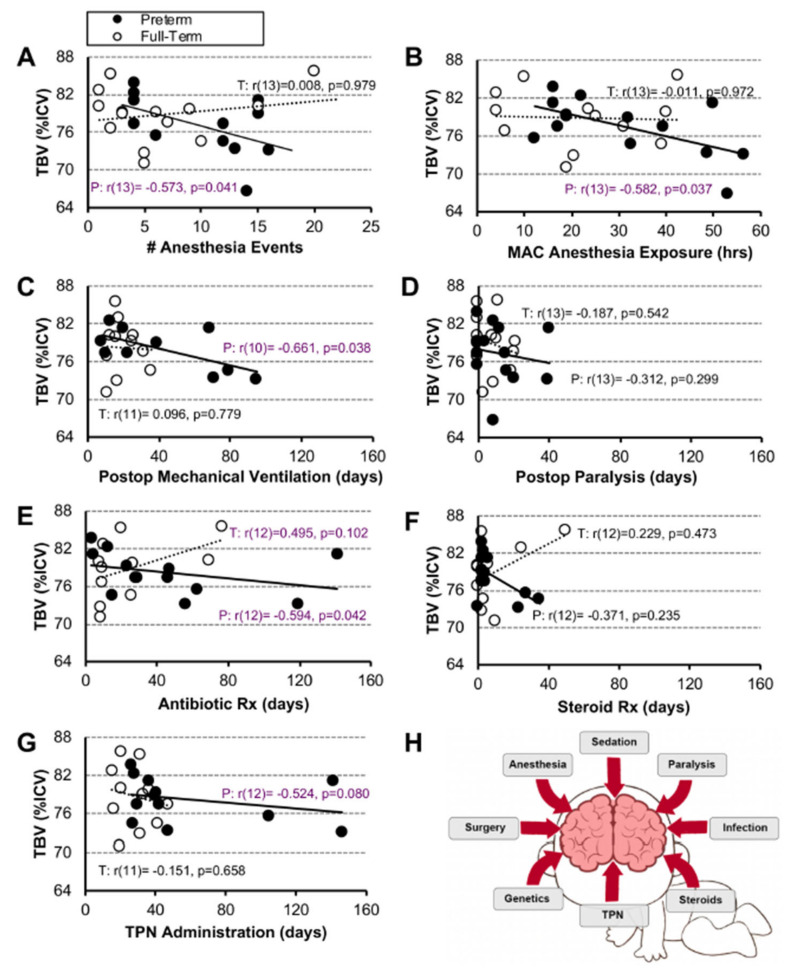
**Association Between Clinical Measures and Normalized Total Brain Volume.** Panels (**A**–**H**) illustrate associations between 7 clinical care measures and normalized total brain volume (TBV as % intracranial volume (ICV)) for early-to-late preterm (n = 10–13; black circles, solid trend line) and term-born infants (n = 11–13; open circles, dashed trend line) following long-gap esophageal atresia (LGEA) repair. We report no significant associations. All correlations were assessed by nonparametric Spearman Rho tests, which are resistant to the effects of outliers. Data were non-normally distributed for clinical measures in Panels (**D**–**G**). Strength of correlation is described as weak (r < 0.4; black), moderate (r ≥ 0.4 to <0.7; purple), or strong, (r ≥ 0.7) with statistical significance as *p* < 0.01 (2-tailed). *Abbreviations*: **#**, number; MAC, minimal alveolar concentration; P, premature; Postop, post-operative; Rx, treatment; T, term-born; TBV; total brain volume; TPN, total parenteral nutrition.

**Normalized Brain Volume.** Having previously shown smaller absolute and normalized total brain volumes [10,20], and potentially delayed brain growth in infants born with LGEA [10,18,20], we examined the relationship between listed clinical variables and normalized brain size. We report that only in the premature infant group, longer exposure to anesthesia (Figure 5A,B) and duration of postoperative clinical care measures (in days; Figure 5C–G) showed moderate and mild negative associations to brain size, respectively, none of which were significant. For full statistical details, see Figure 5.

**Figure 6:**
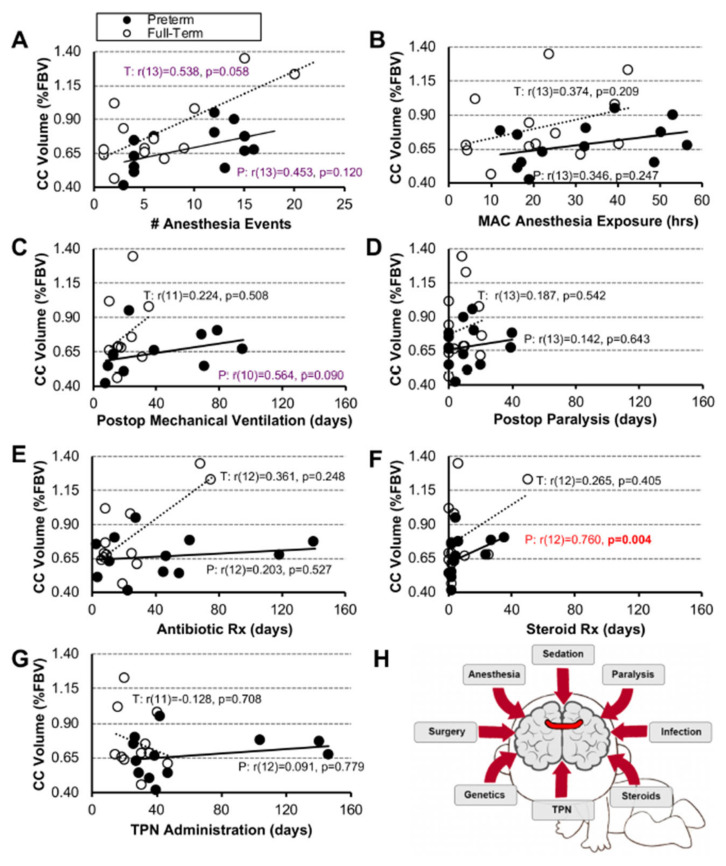
**Association Between Clinical Measures and Normalized Corpus Callosum Size**. Panels (**A**–**H**) show associations between 7 clinical care measures and normalized corpus callosum (CC) volume (as % forebrain volume (FBV)) for early-to-late preterm (n = 10–13; black circles, solid trend line) and term-born infants (n = 11–13; open circles, dashed trend line) following long-gap esophageal atresia (LGEA) repair. All correlations were assessed by nonparametric Spearman Rho tests, which are resistant to the effects of outliers. Data were non-normally distributed for clinical measures in Panels (**D**–**G**). Strength of correlation is described as weak (r < 0.4; black), moderate (r ≥ 0.4 to <0.7; purple), or strong, (r ≥ 0.7; red) with statistical significance as *p* < 0.01 (2-tailed). *Abbreviations*: **#**, number; MAC, minimal alveolar concentration; P, premature; Postop, post-operative; Rx, treatment; T, term-born; TPN, total parenteral nutrition.

**Normalized Corpus Callosum Volume.** We previously reported disproportionally smaller normalized corpus callosum (CC) volumes [19] in a pilot cohort analyzed following complex perioperative critical care with the Foker process for LGEA repair. While one would expect that longer exposure to clinical metrics would be associated with smaller normalized CC volumes, we see the opposite trend. All clinical variables were positively associated with normalized CC volumes, with significant positive association between length of steroid treatment in premature infants (r(12) = 0.760, *p* = 0.004; Figure 6F). This reversal may be due to the cohort’s small sample size, the small normalized CC values (in a range within 1% of forebrain volume), and the small scale of group differences, which all warrant further investigation and should be interpreted with caution. For full statistical details, see Figure 6.

### 3.4. Multivariable Linear Regression Models

We performed multivariable regression models that included group status and six clinical end-point measures (Table 2), with length of TPN administration excluded (see Methods section). We report that listed variables *together* significantly predicted the number of cranial MRI findings (F(6, 14) = 3.12, *p* = 0.037), but not total brain (F(6, 14) = 1.11, *p* = 0.405) or corpus callosum volumes F(6, 14) = 0.99, *p* = 0.655) for both term-born and early-to-late premature patient groups. Interestingly, none of the individual variables studied significantly predicted the number of cranial MRI findings, total brain volume, or total CC volume on their own. For full statistical details, see Table 2.

## 4. Discussion

This report assessed disease severity scores and easily quantifiable clinical measures as potential early markers of qualitative [18] and quantitative [10,20] brain MRI findings in a pilot group of infants following repair of LGEA with the Foker process [7]. Although individual clinical parameters were of limited use in predicting brain findings on their own, *together,* they may serve as an early indirect indicator of possible neurological risk.

### 4.1. Underlying Disease Severity Scoring Metrics’ Validity for Assessing Brain Findings following Long-Gap Esophageal Atresia Repair

Although critical illness in infancy has been known to be associated with neurocognitive morbidities [35], our report represents the first attempt to assess the relationship between ASA classification and PRAm scores with cranial MRI findings. Our study failed to show any association between either ASA or PRAm scores and cranial MRI findings for either term-born or early-to-late preterm infants (Figure 2). Furthermore, our results indicate that PRAm scoring may be of limited use in the selected cohort of infants born with LGEA, despite being introduced as a novel scoring system specifically designed for predicting perioperative risk of mortality in pediatric populations undergoing noncardiac surgery [36]. In contrast, ASA classification supports that premature infants were more critically ill in comparison to term-born infants in our pilot cohort. Indeed, it is widely known that prematurity is a confounding factor in critical illness (with various morbidities) [37], including neurologic and neurocognitive sequelae [38], but future studies are needed to rule out prematurity in relation to brain findings in the context of complex perioperative critical care as part of the Foker process [7,8,9]. The lack of association between ASA classification and brain findings, despite documented incidental clinically significant MRI findings [10] and smaller brain and CC volumes [10,18,20], probes for (i) a study with larger power and/or (ii) a future new scoring metric in order to expand on previous risk stratification in this unique patient population. Future studies should also explore the usefulness of other scoring tools (viz. pediatric risk of mortality score (PRISM) [39], pediatric logistic organ dysfunction score (PELOD) [40], and pediatric multiple organ dysfunction score (P-MODS) [41] in assessing risk of brain findings.

### 4.2. Validity of Individual and Combined Clinical Parameters as Predictors of Brain Findings

Most of our *individual* clinical metrics (Figure 4, Figure 5 and Figure 6) showed no significant associations with brain MRI measures. In contrast, using a multivariable linear regression model, we report that the group status and clinical end-point measures *together* play a role relating to the number of incidental cranial MRI findings (Table 2), irrespective of the gestational age. Similarly, previous studies in premature infants found that a combination of stressful/painful events during neonatal critical care, as well as the interaction between underlying disease and therapeutic interventions, may, *together,* contribute to an allostatic load [42] and possibly poorer health outcomes [43]. Future studies should attempt to evaluate combined clinical parameters, along with other measures of care, to better understand intrinsic disease and treatment impact for life-saving LGEA repair with the Foker process [7]. Of all the clinical measures, easily quantifiable cumulative MAC anesthesia exposure (hrs) had a *p* = 0.05 value (Table 2), which warrants future studies with larger power before it could be considered a possible early individual indirect marker for risk of brain findings. This implication is in line with previous reports that have established quantification of individual repeated anesthetic exposure in infancy as a possible predictor of neurological outcomes later in childhood [44,45,46]. Thus, future multicenter studies could help validate the use of anesthesia quantification as a possible early indirect marker of brain vulnerability in the context of complex perioperative critical care in infancy.

### 4.3. Long-Term Evaluation and Neurodevelopmental Outcomes

There is a gap in our knowledge with respect to long-term neurodevelopmental outcomes of infants born with LGEA that underwent Foker process repair, as most reports focus only on surgical outcomes in the cohort of interest [25,47,48,49]. Our most recent retrospective analysis [50] showed increased survival of infants born with esophageal atresia when compared to previous decades (using Spits [51] and Waterson’s [52] evaluation), highlighting the need for this line of inquiry. The most recent meta-analysis of the literature reported conflicting results regarding the long-term neurodevelopment in children born with esophageal atresia [53]. Only two recent reports implicate neurologic findings and increased risk of brain injuries and long-term neurodevelopmental sequalae in a cohort of infants born with gastrointestinal anomalies that included esophageal atresia (without distinction with respect to surgical type: short-gap vs. long-gap) [16,17].

Two landmark multicenter prospective trials, GAS [54] and RESTORE [55], evaluated long-term neurodevelopmental outcome following anesthesia exposure and prolonged sedation, respectively. However, the GAS study reported no negative neurodevelopmental outcomes in otherwise healthy (ASA I and II) infants following simple hernia repair at 2- [56] or 5-year [54] follow-up. Similarly, results from the RESTORE study [55] do not apply to our cohort of infants born with LGEA exposed to prolonged sedation, since their subjects (i) underwent sedation for treatment of primary respiratory disease in the absence of surgery, and (ii) had a smaller mean length of sedation of 6.5 days.

Very importantly, implication of early illness associated with intraoperative hypotension, hypoxemia, and anemia has been shown to increase the risk of morbidity and mortality early in life, as demonstrated by a recent prospective observational study (NECTARINE trial; [57]). Similarly, perioperative periods of diminished cerebral oxygen delivery are associated with abnormalities in Psychomotor Developmental Index and brain magnetic resonance findings in infants undergoing reparative heart surgery [58]. Although our cohort did not include any LGEA infants undergoing cardiac surgeries, it is well known that about 50% of infants with LGEA have co-existing congenital heart disease [50], and that children supported on extra-corporeal membrane oxygenation (ECMO) for cardiac indications have significant developmental delays and warrant close neurodevelopmental follow-up [59]. Furthermore, early postoperative brain volumes are associated with one-year neurodevelopmental outcome in children with severe congenital heart disease [60]. Long-term health-related quality of life is diminished in children following neonatal surgery for congenital heart abnormalities, with specific deficits in school functioning, intelligence quotients, and neuromotor abilities [61,62,63,64]. Moreover, the length of mechanical ventilation and the length of exposure to sedative and analgesic drugs have been negatively associated with quality-of-life findings at 12-month and 4-year follow-up [65,66,67]. Future studies in infants born with LGEA should also explore the impact of brain perfusion and possible poor tissue oxygenation as additional clinical markers. Our pilot findings call for both early- and long-term neurobehavioral follow-up after complex perioperative critical care for LGEA repair, both in the absence and presence of congenital heart disease or syndromes.

### 4.4. Study Limitations

Since the correlation and regression analyses used in this study measure the strength of association between two selected variables without insight into etiology, future studies are needed to elucidate the underlying mechanisms of previously presented brain findings [10,19,20,21]. Additionally, several study limitations should be noted: *Sex Differences.* Our term-born and premature patient groups had an even sex distribution, which is reflective of the relatively equal sex distribution of LGEA infants, as reported by recent retrospective analysis from *The Esophageal and Airway Treatment Center* at our institution [50]. However, potential sex differences were not analyzed due to lack of power. *Study Size*. Future studies should include at least 16 subjects/sex/gestational group to detect a change of 0.25. These power calculations [68,69,70] are in accordance with infant studies of volumetric analyses (n = 11–13/group) [71] and long-term neurodevelopmental outcomes (n = 13–16/group) [72]. *Timing of MRI scans.* Scans were collected throughout the 1st year of life, leading to a non-uniform time difference between completion of treatment and research MRI scans, introducing potential bias. Furthermore, MRI scans were not collected prior to Foker process treatment, so it is impossible to assess preexisting differences in brain findings or refute the possibility that detected alterations were associated with prematurity alone and not the caregiving conditions associated with the complex care for LGEA repair. *Estimation of quantification.* Some of the infants were transferred from another institutions, likely resulting in underestimation of anesthesia exposure for procedures performed elsewhere. Future multicenter studies could overcome this limitation. As anesthetic, analgesic, and sedative administration was not protocolized at our institution, each infant received a slightly unique combination of agents that also introduces bias.

## 5. Conclusions

Reported individual associations do not represent a causative relationship, and prematurity should be considered a confounder, as premature infants are known to be sicker. Gestational age grouping with the easily quantifiable listed clinical end-point measures could be combined for assessing the early impact of allostatic load on brain findings following LGEA repair. The impact of complex perioperative critical care with prolonged sedation in the context of LGEA repair with Foker process calls for future studies of long-term neurodevelopmental outcomes in both early-to-late premature and term-born infants.

## Figures and Tables

**Table 1:** Qualitative MRI findings.

Qualitative MRI Findings	# PRETERM (n = 13)	# TERM-BORN (n = 13)	Total # Anomalies
***Brain Abnormalities***			
Increased extra-axial space	11	7	18
Widened sylvian fissures	5	5	10
Enlarged/Prominent Ventricles	11	9	20
Low cerebellar volume	0	0	0
Low brainstem volume	0	0	0
Chronic blood products (e.g., hemosiderin)	0	2	2
Mass or cyst	1	2	3
Narrowing of cerebral aqueduct	1	0	1
Incomplete rotation of hippocampi	2	0	2
***White Matter Abnormalities***			
Low cerebral white matter volume	1	0	1
Abnormal signal in white matter	2	4	6
Corpus callosum abnormalities	12	10	22
***Grey Matter Abnormalities***			
Low cerebral grey matter volume	0	1	1
Abnormal signal in grey matter	0	0	0
***Vasculature Abnormalities/Hemorrhage***			
Intraventricular Hemorrhage	1	0	1
Subdural hemorrhage	3	0	3
Subdural effusion/collection	1	2	3
Arterial ischemic/hemorrhagic stroke	1	0	1
Venous hemorrhagic stroke	0	1	1
Cerebellar hemorrhage (arterial or venous) stroke	0	1	1
Vascular anomaly	1	0	1
Possible parturitional hemorrhage	0	1	1
***Cranial Abnormalities***			
Abnormal head shape (e.g., plagiocelphaly)	3	2	5
Non-CNS cranial anomaly	3	2	5

Table 1 summarizes the type and incidence of clinically relevant cranial MRI findings in the cohort of early-to-late premature and term-born infant patients (n = 13/group). The number (#) of each qualitative MRI finding is totaled in the right column. Individual infants had more than one finding listed. For the individual number of cranial MRI findings/patient, refer to Figure 1A. *Abbreviations:* CNS, central nervous system.

**Table 2:** Multivariable model for number of cranial MRI findings: regression analysis.

Clinical Variables	Regression Coefficient	95% CI	*p* Value
**1. Group Status (Preterm vs. Term-born)**	0.026	−1.881 to 1.932	0.98
**2. MAC Anesthesia Exposure (hrs)**	0.119	0.002 to 0.237	0.05
**3. Intubated Sedation (days)**	0.031	−0.05 to 0.113	0.42
**4. Postoperative Paralysis (days)**	−0.014	−0.157 to 0.128	0.83
**5. Antibiotic Rx (days)**	−0.029	−0.064 to 0.007	0.11
**6. Steroid Rx (days)**	−0.039	−0.166 to 0.088	0.52

Multivariable linear regression model showed that listed 6 clinical end-point measures *together* significantly predicted the number of cranial MRI findings for both early-to-late premature and term-born infant infants following long-gap esophageal atresia (LGEA) repair (F(6, 14) = 3.121, *p* = 0.037), but none of the *individual* measures did on their own (right column *p* values). *Abbreviations*: hrs, hours; MAC, minimal alveolar concentration; Rx, treatment.

## Data Availability

The unidentified raw data supporting the conclusions of this article will be made available upon request to the corresponding author.

## Abbreviations

ASA	American Society of Anesthesiologists
LGEA	long-gap esophageal atresia
MAC	minimal alveolar concentration
MRI	magnetic resonance imaging
PRAm	Pediatric Risk Assessment
r	Spearman Rho test
SGA/IUGR	small for gestational age/intrauterine growth restriction
TEF	tracheo-esophageal fistula
TIVA	total intravenous anesthesia
TPN	total parenteral nutrition
VIF	variance inflation factor
